# Development and Validation of a Questionnaire to Measure Adherence to the Mediterranean Diet in Korean Adults

**DOI:** 10.3390/nu12041102

**Published:** 2020-04-16

**Authors:** Yu-Jin Kwon, Hyangkyu Lee, Yooeun Yoon, Hyung Mi Kim, Sang Hui Chu, Ji-Won Lee

**Affiliations:** 1 Department of Family Medicine, Yongin Severance Hospital, Yonsei University College of Medicine, Seoul 16995, Korea; digda3@yuhs.ac; 2 Department of Nursing, Mo-Im Kim Nursing Research Institute, Yonsei University College of Nursing, 50-1, Yonsei-ro, Seodaemun-gu, Seoul 03722, Korea; HKYULEE@yuhs.ac; 3 Department of Nutrition and Dietetics, Gangnam Severance Hospital, Seoul 135-720, Korea; yye1214@yuhs.ac; 4 Department of food and nutrition, Dongduck Women’s University, Seoul 02748, Korea; veronykim@naver.com; 5 Department of Family Medicine, Yonsei University College of Medicine, Gangnam Severance Hospital, 211 Eonju-ro, Gangnam-gu, Seoul 06273, Korea

**Keywords:** Mediterranean diet, adherence, questionnaire, lipids

## Abstract

The Mediterranean diet (MD) has beneficial effects on cardiovascular diseases, cancer, and mortality. Although various attempts have been made for estimating adherence to the MD using diet quality indices, few studies involving validated questionnaires for estimating adherence have been performed in Asian populations. We aimed to develop and validate the Korean version of the Mediterranean Diet Adherence Screener (K-MEDAS) by including 211 participants that visited health check-up centers and 116 participants with overweight or hypercholesterolemia that visited obesity clinic. The participants completed both the K-MEDAS and a 106-item food frequency questionnaire (FFQ). We translated 13 questions and developed 1 question. Considering the agreement between the K-MEDAS and FFQ, nine of the 14 questions showed moderate or high kappa values (≥0.4). The total MD scores measured by the K-MEDAS and FFQ showed substantial concordance (intraclass correlation coefficient = 0.678, 95% confidence interval: 0.520, 0.785). Multiple linear regression analyses revealed significant inverse associations between MD score and the levels of serum total cholesterol, low-density lipoprotein cholesterol, and non-high-density lipoprotein cholesterol, after adjusting for confounding variables. We found that K-MEDAS is valid tool for assessing adherence to the MD in the Korean population.

## 1. Introduction

The Mediterranean diet (MD), characterized by high consumption of plant-based food (vegetables, fruits, whole grains, legumes, nuts, and olive oil), and low consumption of animal-based food, has been validated for its beneficial health effects [1]. Many clinical trials and observational studies have established that MD adherence reduces cardiovascular diseases (CVD) risk, cancer, and mortality [2,3,4,5]. Although the exact mechanism of the MD’s favorable effects is not known, accumulating evidence indicates that its most important roles include a lipid-lowering effect, protection against inflammation and oxidative stress, modification of hormones and growth factors involved in the pathogenesis of cancer, and gut microbiota-mediated production of metabolites influencing metabolic health [6].

Over the years, various attempts for estimating adherence to the MD have been made with the use of diet quality indices. The association of an 8-item Mediterranean dietary score developed by Trichopoulou et al. with several health outcomes, such as all-cause mortality, has been assessed [7]. The Spanish European Prospective Investigation into Cancer and Nutrition (EPIC) study revealed that the relative Mediterranean diet score (rMED), which consisted of nine items and a maximum score of 18, was associated with a reduced incidence of coronary heart disease (CHD) [8]. In 2011, the PREvención con DIeta MEDiterránea (PREDIMED) study, which is the largest trial to investigate the effectiveness of the MD for primary prevention of CVD in high-risk participants, validated a 14-point Mediterranean Diet Adherence Screener (MEDAS). The MEDAS is a well-validated tool for assessing MD adherence in large population-based studies (intraclass correlation coefficient = 0.51) [9].

Since eating habits differ across countries and races, it is difficult to apply the existing MEDAS to other populations. Verification of the questionnaire is needed for its use in other races and countries. Therefore, a German study developed and validated the German version of the MEDAS questionnaire [10], and Papadaki et al. assessed the accuracy and reliability of the English version of the MEDAS in a total of 96 adults with high CVD risk in the United Kingdom (UK) [11]. However, few studies have been conducted using validated questionnaires for estimating adherence to the MD in Asian populations [12,13].

The traditional Korean diet pattern is characterized by high consumption of whole grain rice, low consumption of animal fat, and high consumption of vegetables [14], and several components of the Korean diet are similar to those of the MD (e.g., high consumption of plant food (fruits, vegetables, beans, and seeds) and low intake of red and processed meat). Due to the nature of a traditional Korean diet, the prevalence of obesity is relatively lower in South Korea than that in Western countries [15]. However, with rapid economic growth and the introduction of Western culture, the prevalence rate of hypercholesterolemia in adults aged above 30 years in South Korea has risen consistently up to 20%, and the prevalence rate of dyslipidemia has increased to 40.5% [16]. These trends have led to an increase in obesity and CVD.

Therefore, we aimed to develop the Korean version of MEDAS (K-MEDAS) modifying the original MEDAS to easily apply to Korean population and validate the K-MEDAS in the general population and a high-risk obese or dyslipidemia population.

## 2. Materials and Methods

### 2.1. Study Population

From July 2019 to December 2019, we recruited a total of 211 participants that visited the Severance check-up center for health check-ups and volunteered to be part of the study. The validation study included 211 (95 men and 116 women) participants that completed both dietary questionnaires (the Korean version of MEDAS (K-MEDAS) and the food frequency questionnaire (FFQ) developed by the Korean Genome and Epidemiology Study). To find the associations between K-MEDAS and lipid profiles, 116 participants with overweight (body mass index ≥27 kg/m^2^) with at least one weight-related complication (e.g., diabetes, prediabetes, hypertension, dyslipidemia, or metabolic syndrome) [17] or hypercholesterolemia (total cholesterol ≥200 mg/dl) that visited the obesity clinic in Yongin Severance hospital were included in the study from July 2019 to November 2019. We classified these participants into the high-risk obesity or dyslipidemia group. This high-risk obesity or dyslipidemia group only completed the K-MEDAS. All participants provided informed consent and this study was approved by the Institutional Review Boards of Gangnam Severance Hospital (IRB No, 3-2019-0139) and Yongin Severance Hospital (IRB No, 9-2019-0005). This study was performed in compliance with the Declaration of Helsinki.

### 2.2. Development of the Korean Version of the Mediterranean Diet Adherence Screener

The study group developed a 14-point score of adherence to the MD (www.predimed.es) [9,18]. We received permission from the authors for the use of the 14-item MEDAS via e-mail. The MEDAS was translated into Korean and reviewed by two physicians and two nutritionists. We translated 13 questions into Korean and developed 1 question. One question (“How many times per week do you consume boiled vegetables, pasta, rice, or other dishes with a sauce containing tomato, garlic, onion, or leeks sautéed in olive oil?”) was difficult to apply to a Korean population. Therefore, we developed the following question based on the opinion of experts group including dietician and physician: “How many times do you consume whole grains (multi-grain rice, rye bread, etc.) per week?”. It was reviewed by two independent Korean translators. It was then translated back to English by two native speakers, who were not part of the study. An expert committee including physicians, nutritionists, and a methodologist agreed to produce a piloted version of the translated questionnaire. Cognitive interviews were conducted with a small sample (20 adults) to examine whether the respondents’ interpretations were consistent with the intended meanings. This process was repeated thrice before finalizing the final translated version of the questionnaire. Fourteen experts evaluated content validity. Following this, the final version of the adapted questionnaire was read and approved by the study team.

### 2.3. Lifestyles and Dietary Assessment

Questions on lifestyle were asked using a self-reported questionnaire. Smoking status was classified as non-smoker, ex-smoker, and current smoker. Alcohol consumption was defined as current alcohol consumption. Physical activity was assessed using an International Physical Activity Questionnaire and measured as metabolic equivalent of task (MET) [19,20]. The K-MEDAS consisted of 14 questions. Each question was scored 0 or 1. One point was given for using perilla oil or olive oil as the principal source of fat for cooking (question; Q1) and for preferring white meat over red meat (Q13). One point was given in the following cases: for Q2, ≥3 teaspoons of perilla oil or olive oil per day; for Q3, ≥2 servings of vegetables per day (1 serving = 1 cup (about 200 mL)); for Q4, ≥1 pieces of fruit per day; for Q5, <1 serving of red meat or sausages per day (1 serving = 100 g); for Q6, <1 serving of butter, margarine, or cream per day (1 serving = 1 teaspoon); for Q7, <1 serving of sugar-sweetened beverages per day (1 serving = 1 cup (100 mL)); for Q8, ≥7 servings of wine per week (1 serving = 1 glass (100 mL)); for Q9, ≥3 servings of beans or tofu per week (1serving = 150g of tofu); for Q10, ≥3 servings of fish or seafood per week (1serving =150g); for Q11, <3 times of sweets, bread (except whole wheat bread), cakes, and cookies per week; for Q12, ≥3 times of nuts per week; and for Q14, ≥3 times of whole grain per week. If the criterion was not met, 0 point was allotted; therefore, the sum of the K-MEDAS score ranged from 0 to 14 points.

All 211 participants were asked to complete a 106-item FFQ previously developed by the Korean Genome and Epidemiology Study (KoGES) [21]. The FFQ used in the KoGES presented a list of foods that Koreans frequently consume and investigated the frequency and quantity of consumption over the past year. Foods were grouped to obtain a corresponding score to the K-MEDAS. All participants were asked to complete the 3-day 24-h dietary recall survey through a web-based application. The nutrition intakes were calculated using a CAN-Pro 5.0 developed by Korean Nutrition Society (http://www.kns.or.kr). The averages of nutrition intakes of the three days were recorded. The proportion of carbohydrate and protein were calculated as carbohydrate intake (g) × 4 kcal/total energy intake (kcal/day), and protein intake (g) × 4 kcal/total energy intake (kcal/day). The proportion of fats (fat, saturated fat, monounsaturated fat, polyunsaturated fat, omega-3, omega-6) were calculated as each fat intake (g) × 9 kcal/total energy intake (kcal/day). The measurement units of vitamins were recorded based on the 9th revision Korean food composition table.

### 2.4. Anthropometric and Laboratory Measurements

Body mass index (BMI) was calculated based on weight in kilograms and height in meters. Adults with a BMI greater than or equal to 25 kg/m^2^ were considered overweight and those with a BMI greater than or equal to 30 kg/m^2^ were considered obese [17]. According to the American College of Cardiology (ACC) and the American Heart Association (AHA) guideline, adults with a BMI greater than or equal to 27 kg/m^2^ with comorbidity and those with a BMI greater than or equal to 30 kg/m^2^ were recommended a comprehensive lifestyle intervention and adjuvant therapies such as pharmacotherapy [17]. Waist circumference (WC) was measured at the level of the mid-point between the inferior margin of the last rib and the iliac crest using a constant tension tape while standing. Blood pressure was checked twice in the sitting position after 5 min of rest. Blood samples were obtained after overnight fasting. Fasting serum glucose, total cholesterol (TC), triglyceride (TG), high-density lipoprotein cholesterol (HDL-C), and low-density lipoprotein cholesterol (LDL-C) levels were measured with the ADVIA 1800 Clinical Chemistry System (Siemens Healthcare Diagnostic, Inc, Tarrytown, NY, USA). Non-HDL-C levels were calculated by deducting the HDL-C level from the TC level.

### 2.5. Statistical Analysis

Data are presented as mean ± standard deviations (SD) or number (%). We identified the FFQ items corresponding to the K-MEDAS. As binary scores were ascribed to each K-MEDAS question, absolute agreement was investigated using a Cohen’s kappa coefficient (κ). The intraclass correlation coefficient (ICC) was used to calculate absolute agreement between the total MD scores measured by the K-MEDAS and FFQ. The agreement between the total score from the K-MEDAS questionnaire and the equivalent FFQ questions was examined using a Bland–Altman analysis. The Bland–Altman analysis is a way to evaluate a bias between mean differences and to estimate an agreement interval, which includes 95% of the differences. We divided the MD score into tertiles (T); T1, ≤5; T2, 6–7; and T3, ≥8. Clinical characteristics and nutritional status of study population according to MD score tertiles were compared using a one-way analysis of variance or chi-square test. Bonferroni correction was performed for multiple comparisons. Furthermore, we carried out multiple linear regression analysis to examine the association between MD score and lipid profiles after adjusting for age, sex, BMI, smoking, alcohol intake, and physical activity (in METs). We also performed multiple linear regression analysis to find the association between MD score and lipid profiles in the high-risk obesity or dyslipidemia group.

All *p*-values were based on two-sided tests, and *p*-values below 0.05 were regarded as statistically significant. We conducted all analyses using SPSS version 25.0 statistical software (SPSS version 23.0; IBM Corp., Armonk, NY, USA).

## 3. Results

Table 1 shows the clinical characteristics of study population according to MD score tertiles. This study included 116 women and 95 men. The mean ± SD of age and MD score were 47.4 ± 10.0 and 6.2 ± 2.2, respectively. Age and level of physical activity were significantly higher in the highest MD score tertile. Of the total population, 144 (68.2%) participants were normal weight, 55 (26.1%) were overweight, and 12 (5.7%) were obese. The status of BMI was not different across the MD score tertiles. Total energy intake was not significantly different among the MD score tertiles. The intake levels of fiber, omega-3 (ω-3), ω-6/ω-3, vitamin C, vitamin E, folic acid, and β-carotene were significantly higher according to increasing MD score tertiles. Regarding the proportion of polyunsaturated fatty acids (PUFA) intake, PUFA (%) was significantly higher in the highest tertile (T3) than in the lowest tertile (T1). The proportions of PUFA intake were not different between T1 and T2 and between T2 and T3. The clinical characteristics of the high-risk obesity or dyslipidemia group according to MD score tertiles are described in Table A1.

Table 2 presents the Korean version of the MEDAS (back-translated into English) and the corresponding questions in the FFQ. Values for absolute agreement between the K-MEDAS and FFQ are also presented in Table 2. As questions regarding the frequency of use and amount of perilla oil or olive oil were not asked during the KoGES, we could not calculate the absolute agreement in terms of Question 2. Regarding Question 8 (wine consumption), none of the patients were assigned any points in either the K-MEDAS or FFQ. Based on previous literature [22], Question 12 and 14 showed almost perfect strength of agreement (kappa = 0.876 and 0.851, respectively). Questions 1, 6, 9, and 11 showed substantial strength of agreement (kappa =0.676, 0.664, 0.613, and 0.707, respectively). Question 5 had the lowest kappa value (kappa = 0.194). The concordance between total MD scores measured by the K-MEDAS and FFQ was substantial (ICC = 0.678).

**Figure 1:**
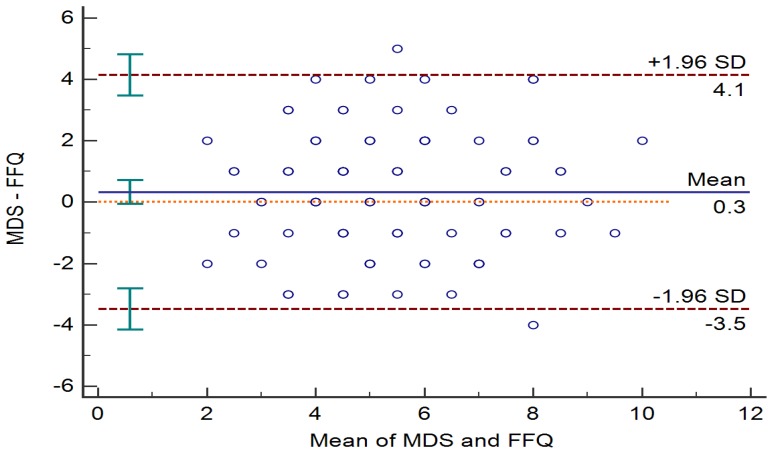
Bland–Altman plots showing the differences between the Korean version of the Mediterranean Diet Adherence Screener score and the food frequency questionnaire score.

The agreement between total MD scores was analyzed using a Bland–Altman plot (Figure 1).

Multiple linear regression analyses revealed significant inverse associations of Mediterranean diet (MD) score with levels of serum total cholesterol (TC), low-density lipoprotein cholesterol (LDL-C), and non-HDL-C after adjusting for age, sex, body mass index (BMI), smoking status, alcohol intake, and physical activity (Table 3). Furthermore, a significant association between MD score and lipid profile was revealed in the high-risk obesity or dyslipidemia group.

## 4. Discussion

We developed and validated a 14 question Korean version of MEDAS adequately modified for Korean diet culture. The serving portions were also tailored to suit Koreans. Nine of the 14 questions showed moderate or high kappa values (≥0.4) between the K-MEDAS and FFQ. The level of absolute agreement between the total MD scores derived from the K-MEDAS and FFQ was moderate (0.678, 95% CI: 0.520, 0.785). These results are comparable to those of previous studies assessing the validation of the MEDAS in Spain, Germany, and the UK [9,10,11]. In addition, the total score of the K-MEDAS was inversely associated with levels of serum TC, LDL cholesterol, and non-HDL-cholesterol in both the general population and the high-risk obesity or dyslipidemia group.

A large amount of evidence indicates that the intake of specific types of nutrients and specific dietary patterns positively influence health and have protective effects against common non-communicable diseases [23]. The MD is the traditional dietary pattern in the Mediterranean region, and it is characterized by a high intake of vegetables, fruits, nuts, legumes, whole grains, and olive oil; moderate consumption of wine, fish, and poultry; and low intake of sweets and red meat [6]. A recent meta-analysis suggested that among all diets evaluated, the MD had the strongest and most consistent evidence of a beneficial effect on cardiometabolic risk factors [1]. Therefore, several countries have developed questionnaires for assessing adherence to the MD.

People from different cultural backgrounds, climates, and environments eat different foods and have different eating habits. Similar to the components of the MD, the traditional Korean diet is characterized by high consumption of grains, vegetables, legumes, and fish, and low consumption of red meat [14]. However, high consumption of white rice and low consumption of fat are the differences between a traditional Korean diet and the MD. No study has developed tools to measure MD adherence based on Korean cultural aspects. Therefore, we developed and validated the K-MEDAS.

We used a semi-quantitative FFQ, which was developed by Korea Centers for Disease Control and Prevention (KCDC) and was used during the KoGES [21]. The KoGES was a large prospective cohort study with government funding. The FFQ involves questions regarding 106 foods frequently consumed by Koreans to survey the frequency of average intake and intake over the past year. In addition, food intake habits, such as the type of oil used in cooking, were surveyed during the KoGES. However, as the oil intake was not measured in the FFQ, we could not calculate the absolute agreement for Question 2 in this study.

Another important feature of the K-MEDAS was the inclusion of questions regarding perilla oil intake, which is consumed in large amounts by Koreans. Olive oil contains high levels of monounsaturated fatty acids (MUFA), especially oleic acid, and other PUFAs; these polyphenols contribute to the reduction in CVD and cancer risks [24]. Traditionally, perilla oil is the main oil used for cooking in Asian countries, including Korea [25]. Perilla oil is a rich source of ω3 PUFAs, specifically alpha-linolenic acid (ALA), and also contains high amounts of ω6 and ω9 fatty acids [26]. Compared to other plant oils, perilla oil has predominant amounts of ω3 fatty acids; therefore, perilla oil is helpful in the prevention of CVD and other inflammatory diseases [26]. Hence, we included the use of perilla oil in Question 1 considering the Korean dietary pattern and the composition of fatty acids in perilla oil.

The MD includes a moderate consumption of red wine. Meanwhile, Koreans usually consume soju (made by distilling alcohol from fermented grains), beer, and rice wines (made by the fermentation of rice starch with yeast). The proportion of subjects drinking a glass of red wine daily was zero in this study. Therefore, we could not calculate the concordance of Question 8.

In the original MEDAS questionnaire, the last question pertained to Mediterranean sofrito (traditional sauce of tomatoes, garlic, onion, and olive oil) [9]. However, sofrito is seldom used in traditional Korean food. In contrast, the Korea population obtains about 65–70% of total energy from dietary carbohydrate, mainly rice [27,28]. Our expert committee deleted the original Question 14 and developed a new question about the consumption of whole grain. It is well known that the type of carbohydrate consumed is important for preventing chronic diseases such as CVD and metabolic syndrome [29]. Whole grain intake has been known to be inversely associated with metabolic risk factors [30]. Therefore, our expert committee decided to include the frequency of whole grain intake in the K-MEDAS.

Dyslipidemia is one of the prominent risk factors of the progression of atherosclerosis, which is an underlying cause of CVD [16]. Emerging evidence revealed that the MD diet has a protective effect against dyslipidemia and CVD [4,31,32,33]. Although the precise mechanism between the MD and lipid metabolism has not yet been elucidated, many interrelated and overlapping components of the MD exert lipid-lowering effects. High consumption of MUFA and omega 3 PUFAs from olive oil, nuts, and fish oil reduce hepatic secretion of triglyceride-rich lipoproteins, LDL cholesterol, and LDL oxidative susceptibility [34,35]. Additionally, substituting saturated fat and trans fatty acids with unsaturated fat lowers the serum LDL cholesterol level [36]. Increased consumption of fiber from whole grains, legumes, vegetables, and fruits also leads to significant reductions in plasma LDL cholesterol levels [37]. Fibers prevent the reabsorption of bile acid in the ileum, therefore, serum cholesterol level are reduced [38]. Lastly, various phytochemicals, such as polyphenols, abundant in vegetables, fruits, and olive oil have anti-oxidant and anti-inflammatory effects that contribute to the prevention of CVD [33]. More large clinical studies are needed to determine whether adherence to the MD or a tailored MD could reduce the prevalence of dyslipidemia and CVD in the Korean population.

Our study has several limitations. First, the study sample was relatively small. Further studies involving a large, representative dataset are needed to validate the usefulness of K-MEDAS. Second, since we conducted this study on participants that visited the health check-up center, we could not administer the K-MEDAS for a second time. Hence, we could not conduct the test–retest for validation. Third, we could not obtain enough information about consumption of red wine. In further studies, additional consideration is needed to replace red wine and to validate the Question 8. Investigation about consumption of grapes, grape juice, cranberries, blueberries, and plums will also be helpful to validate the Question 8. Finally, the proportion of women in the high-risk group was high. As the study recruited people visiting a single obesity center, there could be selection bias. Therefore, further studies among other high-risk groups are necessary. Despite these weaknesses, our study has some strengths. To our knowledge, this is the first study to develop a questionnaire for adherence to the MD reflecting the Korean diet. Moreover, the agreements between the K-MEDAS and FFQ were reliable. We also found that associations between the MD score and lipid profiles in the general population and the high-risk obesity or dyslipidemia group.

## 5. Conclusions

We developed the K-MEDAS as a valid tool for the assessment of adherence to the MD in a Korean population. Large population-based studies to determine the usefulness of K-MEDAS are needed. It could help health care workers to monitor dietary patterns of Korean population and to provide guideline for healthy diet pattern to participants.

## Figures and Tables

**Table 1:** Clinical characteristic of the study population according to the Mediterranean diet (MD) score tertile.

MD Scores	Total	Tertile1 (≤5)	Tertile2 (6–7)	Tertile3 (≥8)
*n*	211	85	63	63
Age	47.4 ± 10.0	43.6 ± 9.1 ^a^	47.6 ± 10.0 ^b^	53.3 ± 8.3 ^c^
Sex, female, *n* (%)	116 (55.0)	41 (48.2)	32 (27.6)	43 (68.3)
Body mass index, kg/m^2^	23.6 ± 3.7	23.4 ± 3.6	24.0 ± 3.9	23.5 ± 3.7
Waist circumference, cm	80.2 ± 10.5	80.1 ± 10.9	82.1 ± 10.3	78.6 ± 10.0
Systolic blood pressure, mmHg	117.9 ± 12.3	116.9 ± 12.7	118.0 ± 12.2	119.2 ± 12.1
Diastolic blood pressure, mmHg	73.5 ± 10.0	72.7 ± 10.4	72.9 ± 9.9	75.2 ± 9.3
Smoking, *n* (%)	66 (31.3)	15 (32.9)	21 (33.3)	17 (27.0)
Alcohol intake, *n* (%)	114 (54.0)	47 (56.0)	40 (63.5)	27 (42.9)
Physical activity, MET	1699.1 ± 1604.3	1435.8 ± 1672.6 ^a^	1624.1 ± 1137.2	2129.2 ± 1829.1 ^c^
Glucose, mg/dl	97.5 ± 17.3	97.4 ± 18.0	97.6 ± 14.7	97.4 ± 19.0
Total cholesterol, mg/dl	200.2 ± 37.6	207.9 ± 32.2	195.2 ± 38.6	194.8 ± 38.6
Triglyceride, mg/dl	109.2 ± 63.0	110.1 ± 58.9	115.1 ± 70.6	101.9 ± 60.4
Low-density lipoprotein cholesterol, mg/dl	119.3 ± 34.8	126.4 ± 37.4	112.6 ± 35.0	108.4 ± 36.5
High-density lipoprotein cholesterol, mg/dl	58.7 ± 13.9	60.0 ± 14.9	56.6 ± 12.3	59.2 ± 14.0
Non-high-density lipoprotein cholesterol, mg/dl	141.5 ± 37.4	147.9 ± 36.4	138.7 ± 38.6	135.7 ± 36.6
Total energy (kcal)	1315.5 ± 488.5	1352.2 ± 506.9	1226.6 ± 488.3	1354.7 ± 458.4
Carbohydrate, %	57.3 ± 9.5	56.3 ± 7.2	57.4 ± 13.7	58.6 ± 6.7
Fiber, g	10.4 ± 6.1	8.9 ± 4.4 ^a^	9.2 ± 5.4 ^b^	13.6 ± 7.5 ^c^
Protein, %	17.7 ± 3.3	17.5 ± 3.4	17.5 ± 3.0	17.7 ± 3.4
Fat, %	25.4 ± 6.8	25.8 ± 6.6	25.7 ± 7.5	24.4 ± 6.2
Saturated fat, %	5.9 ± 2.3	6.3 ± 2.4	5.9 ± 2.1	5.5 ± 2.2
Monounsaturated fatty acid, %	6.6 ± 4.6	6.4 ± 2.1	6.5 ± 2.2	6.6 ± 4.6
Polyunsaturated fatty acid, %	5.4 ± 1.9	5.0 ± 1.8 ^a^	5.3 ± 2.1 ^a,b^	6.1 ± 1.7 ^b^
Omega-6, %	4.6 ± 3.1	4.8 ± 1.6	5.0 ± 5.2	4.8 ± 1.3
Omega-3, %	0.9 ± 0.6	0.7 ± 0.4 ^a^	0.8 ± 0.6 ^b^	1.2 ± 0.8 ^c^
Omega-6/Omega-3	6.9 ± 3.8	7.4 ± 3.2 ^a^	7.6 ± 4.8 ^b^	5.5 ± 2.9 ^c^
Vitamin C, mg	53.1 ± 42.2	48.5 ± 46.3 ^a^	44.5 ± 30.0 ^b^	67.7 ± 43.5 ^c^
Vitamin E, mg	9.2 ± 4.7	8.7 ± 4.3 ^a^	8.4 ± 4.3 ^b^	10.8 ± 5.0 ^c^
Folic acid, µg	164.7 ± 114.4	135.6 ± 77.7 ^a^	147.3 ± 118.6 ^b^	221.3 ± 131.7 ^c^
β-carotene, µg	2499.8 ± 2035.0	1883.0 ± 1252.3 ^a^	2104.0 ± 1437.1 ^b^	3727.7 ± 2761.1 ^c^

MD, Mediterranean diet; MET, metabolic equivalent of task; Data are presented as mean ± standard deviation (SD) or number (%). ^a,b,c^ Modes with different superscripts are significantly different (*p* < 0.05). This result is Bonferroni corrected. *p*-values were calculated using a one-way analysis of variance for continuous variables or a chi-square test for categorical variables.

**Table 2:** Agreement of Korean MEDAS questions and transfer of food intake data from the FFQ.

K-MEDAS Questions	FFQ *	Kappa	ICC
Q1. Do you usually use perilla oil or olive oil when cooking?	1 point given based on use of perilla oil or olive oil when cooking †	0.676 (0.517, 0.783)	N/A
Q2. How much perilla oil or olive oil do you consume per day?	n/a	-	-
Q3. How many vegetables do you eat per day?	1 point given based on FFQ calculation, if ≥3 portions of vegetables per day	0.426 (0.143, 0.615)	N/A
Q4. How many fruits do you eat per day?	1 point given based on FFQ calculation, if ≥2 portions of fruits per day	0.528 (0.296, 0.684)	N/A
Q5. How much of red meat (i.e., beef, pork, etc.) and processed meat (ham, sausage, etc.) do you consume per day?	1 point given based on FFQ calculation, if <2 portions of red meat and processed meats per day	0.194 (−0.203, 0.460)	N/A
Q6. How much butter, margarine, and cream did you consume per day?	1 point given based on FFQ calculation, if <1 portion of butter and margarine per day	0.664 (0.498, 0.775)	N/A
Q7. How many drinks that contain sugar do you drink per day (carbonated drinks, juices, processed drinks, etc.)?	1 point given based on FFQ calculation, if <1 portion of soft drinks per day	0.595 (0.395, 0.728)	N/A
Q8. How much wine do you consume per week?	1 point given based on FFQ calculation, if ≥7 cups of wine per week	N/A	N/A
Q9. How much did you consume beans or tofu per week?	1 point given based on FFQ calculation, if ≥3 portions of beans and tofu per week	0.613 (0.422, 0.740)	N/A
Q10. How much fish or seafood did you consume per week?	1 point given based on FFQ calculation, if ≥3 portions of fish and seafood per week	0.273 (−0.085, 0.513)	N/A
Q11. How many times do you consume sweets (chocolate, candy, ice cream, snacks), breads (except whole wheat bread), cakes, and cookies per week?	1 point given based on FFQ calculation, if <2 times of sweets, cakes, cookies, and breads per week	0.707 (0.563, 0.804)	N/A
Q12. How many times do you consume nut products per week?	1 point given based on FFQ calculation, if ≥3 times of nuts per week	0.876 (0.815, 0.917)	N/A
Q13. Do you have a higher preference to consume white meat (chicken breasts, etc.) rather than red meat (beef, pork, etc.) and processed meat (ham, sausage, etc.)?	1 point given based on FFQ calculation, if consumption frequency of poultry and chicken > those of red meat and processed meat.	0.386 (0.084, 0.589)	N/A
Q14. How many times do you consume whole grains (multi-grain rice, rye bread, etc.) per week?	1 point given based on FFQ calculation, if ≥3 times of whole grains per week	0.851 (0.777, 0.900)	N/A
Total scores		NA	0.678(0.520,0.785)

MEDAS, Mediterranean diet adherence score; K-MEDAS, Korean Mediterranean diet adherence score; FFQ, Food frequency questionnaire; ICC, intraclass correlation coefficient * FFQ was developed by Korean Genome and Epidemiology Study. ^†^ This question was not included in FFQ; however, it was surveyed during the Korean Genome and Epidemiology Study (KoGES). * Kappa values for Question 8 were not calculated as the score for Q8 was zero for all participants (K-MEDAS and FFQ).

**Table 3:** Multiple adjusted regression coefficients and 95% confidence intervalsof the association between the Mediterranean diet score and lipid profiles in the general population and high-risk group.

	General Population				High Risk Group			
Dependent Variables	Beta	95% CI		*p*-Value	Beta	95% CI		*p*-Value
Total cholesterol	−4.838	−0.7482	−2.194	<0.001	−5.640	−10.224	−1.055	0.017
LDL cholesterol	−2.558	−5.584	−0.723	0.011	−5.373	−9.507	−1.239	0.012
Non-HDL-C	−3.993	−6.588	−1.398	0.003	−6.470	−10.915	−2.026	0.005
Triglyceride	−3.417	−7.591	0.757	0.108	−6.641	−14.434	1.152	0.094
HDL-cholesterol	−0.845	−1.701	0.012	0.053	0.830	−0.987	2.648	0.365

CI, confidence interval; LDL, low-density lipoprotein; HDL, high-density lipoprotein. Adjusted for age, sex, BMI, smoking, alcohol intake, physical activity (MET) in general population or physical activity (%) in the high-risk group.

## Appendix A

Clinical characteristic of high-risk group according to the Mediterranean diet (MD) score tertile.

**Table A1:** Clinical characteristic of high-risk group according to the MD score tertile.

MD Scores	Total	Tertile 1 (≤4)	Tertile 2(5–6)	Tertile 3 (≥7)
*n*	116	37	45	34
Age	53.0 ± 10.4	49.9 ± 10.8	54.1 ± 8.1	54.6 ± 12.8
Sex, female, *n* (%)	108 (89.1)	32 (86.4)	41 (91.1)	31 (91.2)
Body mass index, kg/m^2^	26.2 ± 4.4	27.1 ± 5.3	26.0 ± 4.3	25.5 ± 3.7
Waist circumference, cm	87.5 ± 11.0	88.6 ± 11.7	87.4 ± 11.7	86.1 ± 9.6
Systolic blood pressure, mmHg	117.0 ± 14.6	113.9 ± 12.6	119.8 ± 16.9	115.6 ± 13.3
Diastolic blood pressure, mmHg	73.9 ± 10.0	73.9 ± 9.1	74.3 ± 11.0	73.2 ± 10.5
Smoking, *n* (%)	8(6.9)	5 (13.5)	2 (4.9)	0 (-)
Alcohol intake, *n* (%)	48 (41.4)	18 (48.6)	32 (71.1)	20(58.8)
Physical activity, *n* (%)	34 (29.3)	7 (18.9)	11 (24.4)	17 (44.1)
Glucose, mg/dl	105.6 ± 18.0	107.8 ± 23.0	105.4 ± 18.6	103.5 ± 10.7
Total cholesterol, mg/dl	212.5 ± 39.5	214.1 ± 37.5	212.8 ± 44.7	207.3 ± 35.1
Triglyceride, mg/dl	132.3 ± 62.1	137.6 ± 57.3	132.0 ± 64.4	118.5 ± 62.6
Low-density lipoprotein cholesterol, mg/dl	129.5 ± 34.6	131.7 ± 33.3	129.6 ± 39.4	124.9 ± 29.5
High-density lipoprotein cholesterol, mg/dl	57.2 ± 13.2	56.5 ± 13.2	56.5 ± 13.0	59.2 ± 13.9
Non-high-density lipoprotein cholesterol, mg/dl	155.4 ± 38.2	157.6 ± 37.8	156.2 ± 42.9	148.1 ± 32.5
Total energy (kcal)	1545.2 ± 584.6	1596.7 ± 702.3	1544.0 ± 616.0	1458.8 ± 420.7
Carbohydrate, %	57.6 ± 13.0	52.8 ± 14.8	60.5 ± 11.3	60.4 ± 11.8
Fiber, g	20.1 ± 9.6	18.7 ± 10.6	20.1 ± 8.0	21.1 ± 10.2
Protein, %	16.3 ± 4.7	16.9 ± 5.7	15.9 ± 4.0	15.9 ± 4.9
Fat, %	25.0 ± 9.7	27.7 ± 11.3	23.2 ± 8.3	23.4 ± 8.5
Saturated fat, %	5.7 ± 10.8	5.2 ± 4.9	5.5 ± 6.9	3.8 ± 3.0
Monounsaturated fatty acid, %	6.9 ± 7.4	6.9 ± 5.9	7.7 ± 9.5	5.3 ± 3.3
Polyunsaturated fatty acid, %	4.7 ± 3.9	5.6 ± 4.4	4.6 ± 3.8	3.7 ± 2.7
Omega-6, %	2.0 ± 4.0	1.9 ± 2.5	1.6 ± 1.7	1.6 ± 1.7
Omega-3, %	0.9 ± 1.8	0.6 ± 1.1	0.4 ± 0.8	0.6 ± 0.9
Omega-6/Omega-3	10.0 ± 7.2	9.0 ± 6.6	9.8 ± 6.9	11.1 ± 8.1
Vitamin C, mg †	100.6 ± 116.2	0.06 ± 0.08	0.07 ± 0.05	0.08 ± 0.07
Vitamin E, mg †	11.8 ± 9.2	0.008 ± 0.005	0.007 ± 0.003	0.006 ± 0.003
Folic acid, µg †	454.3 ± 230.0	0.28 ± 0.15	0.32 ± 0.13	0.32 ± 0.14
β-carotene, µg †	3390.1 ± 3085.2	1.7 ± 1.2	2.5 ± 2.4	2.6 ± 2.7

† adjusted for total calorie intake.

## References

[1] Magkos F., Tetens I., Bugel S.G., Felby C., Schacht S.R., Hill J.O., Ravussin E., Astrup A. (2020). A Perspective on the Transition to Plant-Based Diets: A Diet Change May Attenuate Climate Change, but Can It Also Attenuate Obesity and Chronic Disease Risk?. Adv. Nutr..

[2] Sofi F., Abbate R., Gensini G.F., Casini A. (2010). Accruing evidence on benefits of adherence to the Mediterranean diet on health: An updated systematic review and meta-analysis. Am. J. Clin. Nutr..

[3] Bellavia A., Tektonidis T.G., Orsini N., Wolk A., Larsson S.C. (2016). Quantifying the benefits of Mediterranean diet in terms of survival. Eur. J. Epidemiol..

[4] Trichopoulou A., Costacou T., Bamia C., Trichopoulos D. (2003). Adherence to a Mediterranean diet and survival in a Greek population. N. Engl. J. Med..

[5] Schwingshackl L., Schwedhelm C., Galbete C., Hoffmann G. (2017). Adherence to Mediterranean Diet and Risk of Cancer: An Updated Systematic Review and Meta-Analysis. Nutrients.

[6] Tosti V., Bertozzi B., Fontana L. (2018). Health Benefits of the Mediterranean Diet: Metabolic and Molecular Mechanisms. J. Gerontol. A Biol. Sci. Med. Sci..

[7] Trichopoulou A., Kouris-Blazos A., Wahlqvist M.L., Gnardellis C., Lagiou P., Polychronopoulos E., Vassilakou T., Lipworth L., Trichopoulos D. (1995). Diet and overall survival in elderly people. BMJ.

[8] Buckland G., Gonzalez C.A., Agudo A., Vilardell M., Berenguer A., Amiano P., Ardanaz E., Arriola L., Barricarte A., Basterretxea M. (2009). Adherence to the Mediterranean diet and risk of coronary heart disease in the Spanish EPIC Cohort Study. Am. J. Epidemiol..

[9] Schroder H., Fito M., Estruch R., Martinez-Gonzalez M.A., Corella D., Salas-Salvado J., Lamuela-Raventos R., Ros E., Salaverria I., Fiol M. (2011). A short screener is valid for assessing Mediterranean diet adherence among older Spanish men and women. J. Nutr..

[10] Hebestreit K., Yahiaoui-Doktor M., Engel C., Vetter W., Siniatchkin M., Erickson N., Halle M., Kiechle M., Bischoff S.C. (2017). Validation of the German version of the Mediterranean Diet Adherence Screener (MEDAS) questionnaire. BMC Cancer.

[11] Papadaki A., Johnson L., Toumpakari Z., England C., Rai M., Toms S., Penfold C., Zazpe I., Martinez-Gonzalez M.A., Feder G. (2018). Validation of the English Version of the 14-Item Mediterranean Diet Adherence Screener of the PREDIMED Study, in People at High Cardiovascular Risk in the UK. Nutrients.

[12] Kanauchi M., Kanauchi K. (2016). Development of a Mediterranean diet score adapted to Japan and its relation to obesity risk. Food Nutr. Res..

[13] Kim Y., Je Y. (2018). A modified Mediterranean diet score is inversely associated with metabolic syndrome in Korean adults. Eur. J. Clin. Nutr..

[14] Song Y., Joung H. (2012). A traditional Korean dietary pattern and metabolic syndrome abnormalities. Nutr. Metab. Cardiovasc. Dis..

[15] Kim S., Moon S., Popkin B.M. (2000). The nutrition transition in South Korea. Am. J. Clin. Nutr..

[16] Rhee E.J., Kim H.C., Kim J.H., Lee E.Y., Kim B.J., Kim E.M., Song Y., Lim J.H., Kim H.J., Choi S. (2019). 2018 Guidelines for the management of dyslipidemia. Korean J. Intern. Med..

[17] Jensen M.D., Ryan D.H., Apovian C.M., Ard J.D., Comuzzie A.G., Donato K.A., Hu F.B., Hubbard V.S., Jakicic J.M., Kushner R.F. (2014). 2013 AHA/ACC/TOS guideline for the management of overweight and obesity in adults: A report of the American College of Cardiology/American Heart Association Task Force on Practice Guidelines and The Obesity Society. Circulation.

[18] Martinez-Gonzalez M.A., Corella D., Salas-Salvado J., Ros E., Covas M.I., Fiol M., Warnberg J., Aros F., Ruiz-Gutierrez V., Lamuela-Raventos R.M. (2012). Cohort profile: Design and methods of the PREDIMED study. Int. J. Epidemiol..

[19] Lee P.H., Macfarlane D.J., Lam T.H., Stewart S.M. (2011). Validity of the International Physical Activity Questionnaire Short Form (IPAQ-SF): A systematic review. Int. J. Behav. Nutr. Phys. Act..

[20] Oh J.Y., Yang Y.J., Kim B.S., Kang J.H. (2007). Validity and Reliability of Korean Version of International Physical Activity Questionnaire (IPAQ) Short Form. J. Korean Acad. Fam. Med..

[21] Kim Y., Han B.G. (2017). Cohort Profile: The Korean Genome and Epidemiology Study (KoGES) Consortium. Int. J. Epidemiol..

[22] Sim J., Wright C.C. (2005). The kappa statistic in reliability studies: Use, interpretation, and sample size requirements. Phys. Ther..

[23] Cena H., Calder P.C. (2020). Defining a Healthy Diet: Evidence for The Role of Contemporary Dietary Patterns in Health and Disease. Nutrients.

[24] Gaforio J.J., Visioli F., Alarcon-de-la-Lastra C., Castaner O., Delgado-Rodriguez M., Fito M., Hernandez A.F., Huertas J.R., Martinez-Gonzalez M.A., Menendez J.A. (2019). Virgin Olive Oil and Health: Summary of the III International Conference on Virgin Olive Oil and Health Consensus Report, JAEN (Spain) 2018. Nutrients.

[25] Pettid M.J. (2008). Korean Cuisine: An Illustrated History.

[26] Asif M. (2011). Health effects of omega-3,6,9 fatty acids: Perilla frutescens is a good example of plant oils. Orient. Pharm. Exp. Med..

[27] Kim H.N., Song S.W. (2019). Association between carbohydrate intake and body composition: The Korean National Health and Nutrition Examination Survey. Nutrition.

[28] Park S.H., Lee K.S., Park H.Y. (2010). Dietary carbohydrate intake is associated with cardiovascular disease risk in Korean: Analysis of the third Korea National Health and Nutrition Examination Survey (KNHANES III). Int. J. Cardiol..

[29] Song S., Lee J.E., Song W.O., Paik H.Y., Song Y. (2014). Carbohydrate intake and refined-grain consumption are associated with metabolic syndrome in the Korean adult population. J. Acad. Nutr. Diet..

[30] McKeown N.M., Meigs J.B., Liu S., Wilson P.W., Jacques P.F. (2002). Whole-grain intake is favorably associated with metabolic risk factors for type 2 diabetes and cardiovascular disease in the Framingham Offspring Study. Am. J. Clin. Nutr..

[31] Shannon O.M., Mendes I., Kochl C., Mazidi M., Ashor A.W., Rubele S., Minihane A.M., Mathers J.C., Siervo M. (2020). Mediterranean Diet Increases Endothelial Function in Adults: A Systematic Review and Meta-Analysis of Randomized Controlled Trials. J. Nutr..

[32] Guasch-Ferre M., Liu G., Li Y., Sampson L., Manson J.E., Salas-Salvado J., Martinez-Gonzalez M.A., Stampfer M.J., Willett W.C., Sun Q. (2020). Olive Oil Consumption and Cardiovascular Risk in U.S. Adults. J. Am. Coll. Cardiol..

[33] Martinez-Gonzalez M.A., Gea A., Ruiz-Canela M. (2019). The Mediterranean Diet and Cardiovascular Health. Circ. Res..

[34] Kris-Etherton P.M., Pearson T.A., Wan Y., Hargrove R.L., Moriarty K., Fishell V., Etherton T.D. (1999). High-monounsaturated fatty acid diets lower both plasma cholesterol and triacylglycerol concentrations. Am. J. Clin. Nutr..

[35] Gill J.M., Brown J.C., Caslake M.J., Wright D.M., Cooney J., Bedford D., Hughes D.A., Stanley J.C., Packard C.J. (2003). Effects of dietary monounsaturated fatty acids on lipoprotein concentrations, compositions, and subfraction distributions and on VLDL apolipoprotein B kinetics: Dose-dependent effects on LDL. Am. J. Clin. Nutr..

[36] Wang D.D., Li Y., Chiuve S.E., Stampfer M.J., Manson J.E., Rimm E.B., Willett W.C., Hu F.B. (2016). Association of Specific Dietary Fats With Total and Cause-Specific Mortality. JAMA Intern. Med..

[37] Salas-Salvado J., Farres X., Luque X., Narejos S., Borrell M., Basora J., Anguera A., Torres F., Bullo M., Balanza R. (2008). Effect of two doses of a mixture of soluble fibres on body weight and metabolic variables in overweight or obese patients: A randomised trial. Br. J. Nutr..

[38] Naumann S., Schweiggert-Weisz U., Bader-Mittermaier S., Haller D., Eisner P. (2018). Differentiation of Adsorptive and Viscous Effects of Dietary Fibres on Bile Acid Release by Means of In Vitro Digestion and Dialysis. Int. J. Mol. Sci..

